# Incorporating basic needs to reconcile poverty and ecosystem services

**DOI:** 10.1111/cobi.13209

**Published:** 2018-11-20

**Authors:** Tomas Chaigneau, Sarah Coulthard, Katrina Brown, Tim M. Daw, Björn Schulte‐Herbrüggen

**Affiliations:** ^1^ Environment and Sustainability Institute University of Exeter Penryn Campus Cornwall TR10 9FE U.K.; ^2^ Northumbria University Lipman Building 207, City Campus Newcastle NE1 8S7 U.K.; ^3^ College of Life and Environmental Sciences University of Exeter Amory Building, Rennes Drive Exeter EX4 4RJ U.K.; ^4^ Stockholm Resilience Centre Stockholm University Stockholm SE‐106 91 Sweden

**Keywords:** decision making, ecosystem services, human needs, multidimensional poverty, thresholds, trade‐offs, well‐being indicators, compensaciones, indicadores de bienestar, necesidades humanas, pobreza multidimensional, servicios ambientales, toma de decisiones, umbrales, 生态系统服务, 幸福指标, 人类需求, 利弊权衡, 决策, 阈值, 多维贫困

## Abstract

Conservation managers frequently face the challenge of protecting and sustaining biodiversity without producing detrimental outcomes for (often poor) human populations that depend on ecosystem services for their well‐being. However, mutually beneficial solutions are often elusive and can mask trade‐offs and negative outcomes for people. To deal with such trade‐offs, ecological and social thresholds need to be identified to determine the acceptable solution space for conservation. Although human well‐being as a concept has recently gained prominence, conservationists still lack tools to evaluate how their actions affect it in a given context. We applied the theory of human needs to conservation by building on an extensive historical application of need approaches in international development. In an innovative participatory method that included focus groups and household surveys, we evaluated how human needs are met based on locally relevant thresholds. We then established connections between human needs and ecosystem services through key‐informant focus groups. We applied our method in coastal East Africa to identify households that would not be able to meet their basic needs and to uncover the role of ecosystem services in meeting these. This enabled us to identify how benefits derived from the environment were contributing to meeting basic needs and to consider potential repercussions that could arise through changes to ecosystem service provision. We suggest our approach can help conservationists and planners balance poverty alleviation and biodiversity protection and ensure conservation measures do not, at the very least, cause serious harm to individuals. We further argue it can be used as a basis for monitoring the impacts of conservation on multidimensional poverty.

## Abstract 2

*Article impact statement*: Assessing human needs and their connections with ecosystem services helps balance poverty alleviation and biodiversity protection.

## Abstract (es)

Incorporación de las Necesidades Básicas para Reconciliar a la Pobreza y a los Servicios Ambientales

**Resumen**

Los administradores de la conservación frecuentemente enfrentan el reto de proteger y mantener la biodiversidad sin producir resultados perjudiciales para las poblaciones humanas (comúnmente pobres) que dependen de los servicios ambientales para su bienestar. Sin embargo, las soluciones benéficas para ambos son comúnmente elusivas y pueden cubrir compensaciones y resultados negativos para las personas. Para tratar con dichas compensaciones se requiere la identificación de umbrales ecológicos y sociales para determinar el espacio de solución aceptable para la conservación. Aunque el bienestar humano como concepto ha ganado prominencia recientemente, los conservacionistas carecen de herramientas para evaluar cómo afectan sus acciones en un contexto dado. Aplicamos la teoría de las necesidades humanas a la conservación al basarnos en una aplicación histórica extensiva de estrategias de necesidades en el desarrollo internacional. Evaluamos cómo se cumplen las necesidades humanas con base en umbrales relevantes localmente en un método participativo innovador que incluyó grupos de enfoque y censos de hogares. Después establecimos conexiones entre las necesidades humanas y los servicios ambientales por medio de grupos de enfoque con informantes clave. Aplicamos nuestro método en la costa oriental de África para identificar los hogares que no podrían cumplir con sus necesidades básicas y para descubrir el papel de los servicios ambientales en el cumplimiento de estas necesidades. Esto nos permitió identificar cómo los beneficios derivados del ambiente estaban contribuyendo al cumplimiento de las necesidades básicas y nos permitió considerar las repercusiones potenciales que podrían surgir por medio de cambios en la provisión de los servicios ambientales. Sugerimos que nuestra estrategia puede ayudar a los conservacionistas y a los planificadores a balancear el alivio de la pobreza y la protección de la biodiversidad y a asegurar que las medidas de conservación, como mínimo, no causen daño serio a los individuos. También sustentamos que puede usarse como base para el monitoreo de los impactos de la conservación sobre la pobreza multidimensional.

## 摘要

保护管理者常常面临的一个挑战是如何在不对依赖于生态系统服务而生活的人们 (通常是贫困人群) 产生不利结果的前提下, 保护和维持生物多样性。然而, 双赢的解决方案往往难以实现, 还可能会掩盖人们要面对的利弊权衡和负面影响。为应对这样的权衡, 我们需要找到生态学和社会学的临界值, 以确定保护上可行的解决方案。虽然人类福祉这一概念近期已开始得到重视, 但保护实践者仍缺乏有效工具来评估其行动在特定环境下对人类福祉产生的影响。我们基于国际发展中人类需求方法在历史上的广泛应用, 将人类需求理论应用于保护领域。通过一个包含焦点团体和家庭调查的创新性的参与式方法, 我们评估了在当地特定的临界值下, 人类需求如何得到满足。接下来我们通过关键焦点团体, 建立了人类需求与生态系统服务的联系。这一方法被应用于东非沿海地区, 以找出基本需求得不到满足的家庭, 并揭示生态系统服务在满足人类需求中的作用。这也让我们得以确定从环境中获得的收益怎样帮助人们满足基本需求, 并分析生态系统服务供给变化可能造成的潜在影响。我们提出的方法可以帮助保护实践者和决策者来平衡扶贫工作和生物多样性保护之间的关系, 并确保保护措施至少不会对个人造成严重的负面影响。我们进一步提出, 这个方法可以为监测保护对多维贫困的影响提供基础。**【翻译: 胡怡思; 审校: 聂永刚】**

## Introduction

Poverty and biodiversity loss are 2 of the world's most critical challenges. It is widely accepted that these are linked problems that frequently coincide at various scales (Turner et al. 2012) and that they should be tackled together (Adams et al. 2004). Any vision of sustainable development must recognize that eradicating poverty is inextricably linked to ecological integrity and vice versa (Raworth 2012). As such, it requires that all people have the resources to fulfill their needs but that humanity's use of natural resources does not stress critical Earth system processes. There is therefore a strong imperative for conservation to consider human well‐being to gain legitimacy, improve conservation outcomes, or determine whether interventions are producing positive outcomes for both people and nature (Milner‐Gulland et al. 2014). A growing body of research addressing these issues seeks to better understand how ecosystem services–the benefits humans gain from the environment–could be managed and enhanced to further improve well‐being and alleviate poverty (Fisher et al. 2013). Achieving this involves a difficult balancing act between 2 competing, and often conflicting, objectives: improving people's lives through access to and consumption of natural resources and ensuring ecological health and sustainability of biodiversity in the face of growing human populations and pressures on resources. This balancing act has proved a challenge for conservation, and in practice steering the contribution of ecosystem services toward greater poverty alleviation is riddled with difficulties and limited success. Many potential benefits fail to reach the poorest people and are captured instead by wealthier and more powerfully positioned groups (Thompson & Homewood 2002). This means scenarios in which both conservation and development goals are achieved are elusive (Chaigneau & Brown 2016) and may mask trade‐offs and negative outcomes for the well‐being of particular people (Daw et al. 2015). These clashing development and environmental priorities (Roe & Elliott 2004) find common ground in international policies and rhetoric about sustainability. United Nations Sustainable Development Goals, for example, signal the reemergence of sustainability and development as part of an integrated set of global ambitions (Griggs et al. 2013).

We directly addressed the challenge identified by Palmer Fry et al. (2017) to incorporate locally valid measures of well‐being to assess environmental outcomes and the call by Milner‐Gulland et al. (2014) to develop empirical evidence and tools to apply well‐being concepts that balance local and universal indicators to inform conservation. We built on work seeking to apply concepts such as well‐being and poverty in assessments of the impacts of conservation (Ferraro & Hanauer 2014), but we sought to make specific advances in the field by applying basic‐need measures to identify context‐specific social thresholds.

### Ecological and Social Thresholds

**Figure 1:**
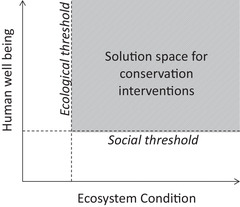
Solution space for conservation interventions based on ecological and social thresholds.

Environmental management is increasingly informed by evidence of nonlinear dynamics in ecosystems and the identification of ecological thresholds. These are points at which environmental degradation or pressures lead to disproportionate and sometimes irreversible environmental change with potentially drastic social and economic effects (Kelly et al. 2015). Although ecological thresholds are increasingly studied, the concept of social thresholds is underrepresented, and we argue that the threshold concept should not be left to the physical sciences alone. We acknowledge that ontological difference means the concept of thresholds does not easily translate across the natural and social sciences, but a threshold point can nevertheless provide a distinct moment that can encourage innovation and transformations in management practice (Christensen & Krogman 2012). Incorporating the concept of a social threshold would improve understanding of points at which impacts become too great to be morally feasible or irrevocable (Walker & Meyers 2004). Combining social and ecological threshold maps provides a potential solution space for morally acceptable conservation interventions, which have the potential to further consensus across affected stakeholders (Fig. 1).

A multidimensional conceptualization of well‐being has been proposed to elucidate the breadth of ways in which ecosystem services can contribute to, or detract from, the quality of people's lives (Milner‐Gulland et al. 2014; Breslow et al. 2016). Conventional understandings of human–environment interactions have been limited by overly narrow interpretations of human welfare, for example, using income or other easily quantified attributes (Coulthard et al. 2018). We argue these narrow interpretations exacerbate the difficulty of navigating trade‐offs between conservation and development objectives.

There are now many frameworks with different criteria that shape how well‐being might be captured, measured, and ultimately understood (Fisher et al. 2013; Breslow et al. 2016). These have helped shift the development debate away from a narrow focus on objective dimensions of poverty, in particular income poverty, to the broader discussion of well‐being (i.e., what people need to be able to have, to be able to do, and be able to feel in order to be well in society) (Gough et al. 2007). As such, different people have different ideas about what is important for their well‐being and how they should seek to achieve it. The fact that different groups of individuals may want different things and have competing interests means that optimizing for conservation or environmental management may not always appear to be the most advantageous for some people (Martin 2017). Where resources are scarce, it is most critical to identify, prioritize, and address situations in which people are deprived of their basic human needs and to focus conservation and development approaches toward addressing the most important deprivations (McGregor et al. 2009). In such instances, the idea of a justifiable minimum social threshold is useful to ensure that no one is left behind in accordance with the UN sustainable development goals.

We propose that such thresholds can be supported by the list of universal criteria for assessing human needs from Doyal and Gough's (1991) theory of human need. The distinctiveness and appeal of this theory over other well‐being or poverty frameworks for informing environmental management and conservation decisions in the face of trade‐offs is 2‐fold. First, it provides a universal list of human needs that apply to all humans on the planet. This is a powerful attribute because it enables a degree of comparability and repeatability and avoids some of the problems of relativism, although the ways in which needs are met are context specific. In her argument for universal lists of well‐being criteria, Nussbaum (2001) argues that such lists can represent “a set of basic constitutional principles that should be respected and implemented by the governments of all nations, as a bare minimum of what respect for human dignity requires.” Second, human needs provide life essentials without which the person would incur serious harm of an objective kind (Doyal & Gough 1991). As such, human needs provide a critical minimum threshold of human welfare that all governments and decision makers could morally respect to maintain in their governed populations. It therefore provides a universal list of criteria that conservationists and decision makers anywhere can agree to adhere to when driven by the principle of do no harm. The theory of human need is one of many approaches applied to conceptualizing poverty and measuring poverty thresholds specifically (Alkire 2002; Tsui 2002), but we argue that its universality and tangibility make it a rich operational framework for addressing hard choices between nature conservation and poverty alleviation goals (Gough 2014; O'Neill et al. 2018) and a basis for monitoring and mitigating conservation impacts on multidimensional poverty.

We devised a novel process to operationalize the human‐needs approach to assess the levels and types of deprivation experienced by people (see also, McGregor et al. 2007). We therefore elaborate on how harm can be conceived and who is being harmed across different circumstances. We applied the methodological approach at eight rural and urban communities in coastal Kenya and northern Mozambique and explored the contribution of ecosystem services in ensuring that people are not in serious harm. Finally, we considered how this approach could help in evaluation of the impact of conservation measures in such a way as to ensure that these measures are not causing people serious harm.

## Method

### Study Context and Sites

The data were collected as part of a larger project (www.espa-spaces.org) working to establish how marine ecosystem services contributed to human well‐being and poverty alleviation in coastal communities in Kenya and northern Mozambique. The study was conducted in 4 sites in Kenya and 4 in Mozambique adjacent to mangrove or coral ecosystems in rural and urban areas (more information on each site is available from www.espa-spaces.org). Community profiles were developed for each site based on secondary sources, participatory observation, and key‐informant interviews. This work identified characteristics of each site and the main livelihood activities, in particular those related to the environment. Urban sites (Kongowea in Mombasa, Kenya, or and Maringanha, a suburb of the city of Pemba in Mozambique) had larger population sizes than other sites and a wider array of livelihood activities. The peri‐urban site of Mieze is along the main road to Pemba in Mozambique, was farther inland than other sites, and agriculture formed the basis of the local economy, although mangroves also supported crab fishing. At the rural site (Mkwiro south of Mombasa, Kenya, on Wasini Island), livelihood activities included tourism (predominantly day trippers from Mombasa) and fishing. At the isolated site of Lalane, Mozambique, north of Pemba, fishing was the primary source of livelihood.

Despite these differences, all communities were deriving some benefits from their adjacent coral reef or mangrove ecosystems. These ecosystems were in different conditions and were managed in different ways. Some sites had no form of conservation or environmental management measures in place (e.g., fisheries in Lalane), whereas others had a nearby managed marine national parks (Kongowea and Mkwiro), a community‐based marine sanctuary supported by a nongovernmental organization and the tourism industry (Vamizi), or mangroves managed through limited licensing by government forest services (Vanga).

To develop a human‐needs approach, we combined expert and community perspectives by enabling public deliberation to evaluate how human needs are met based on locally relevant thresholds. Developing a set of agreed‐on indicators for basic needs, determining the degree to which they are met within communities, and evaluating the contribution of ecosystem services to them were undertaken in 5 distinct steps (Supporting Information).

### Verifying the List of Needs

This first step introduced the theory‐based list of human needs and aimed to ascertain the extent to which the list reflected community conceptualizations of human needs and to capture differences among communities.

In each site, men's and women's focus groups were convened. We conducted 16 focus groups in all (2 at each site). Participants were purposively sampled based on information gathered via community profiling and key‐informant interviews to incorporate a range of income groups, ethnic groups, primary occupations, gender, and geographical areas of the community. Each focus group was asked, “How would you describe a household that is doing well or doing badly?” The emergent list of context‐relevant well‐being criteria was then compared with a list of 12 theory‐derived basic human needs (shelter, economic security, sanitation, drinking water, food security, health, education, physical security, respect, relationships, autonomy, and participation) to ensure they were comparable and avoid missing characteristics of well‐being important to communities. When new aspects of well‐being were mentioned that were not captured, these were added to the list in later steps. To ensure the consistency within sites and a correspondence with the preexisting research and theory on needs, if specific needs were not mentioned by participants, these were still included in the subsequent steps.

### Eliciting Need Indicators

In the second step, within the same focus groups, indicators were elicited for each need that were more specific than those identified in the first step because the second step focused on specific characteristics of each need that could enable their measurement. Whereas needs are considered universal, the ways in which they are satisfied (i.e., whether people are above or below a level at which the need is met [threshold of harm]) may vary in different contexts (Doyal & Gough 1991). For each need therefore, we derived a list of need indicators by asking participants to describe conditions under which a person is doing well or badly for each need.

### Identifying Basic‐Need Thresholds

In the third step, a follow‐up focus group at each site was carried out with a subset of people from each focus group to determine site‐specific thresholds of harm for each need (Supporting Information). The indicators generated in step 2 were grouped together under the different needs, and we asked participants to arrange the list for each need from doing well to serious harm. The participants were then asked to reflect on the ordered list of indicators and for each need deliberate and decide at which point they consider a person or a household to be in serious harm due to deprivation of that need. This was equivalent to a human‐needs threshold, above which a need is met and below which a need is unmet.

### Creating Household Survey Questions

In the fourth step, we took the indicators from step 3 that were close to the threshold of harm (e.g., a person sometimes does not eat for a whole day) and converted these into simple questions for inclusion in a large‐scale household survey (e.g., “Over the last year, have you ever not eaten for a whole day due to lack of food?”). The survey was then administered to a representative sample of the population at each site, and simple data‐processing rules were used to evaluate whether each basic need was met or not for each respondent. The final thresholds and processing rules were based on a triangulation between the contextual information from focus groups and local and expert views. In a few cases, rules also reflected expert judgment where focus group outputs did not fully reflect possible harm (e.g., from polluted water sources).

The household survey was conducted across 1130 randomly selected households. For representation of within‐household variation (Agarwal 1997), we interviewed up to 3 people per household where possible, including the household head, spouse, and a randomly chosen third person over 15 years old, resulting in a total of 2293 interviews. To aggregate multiple responses per household to a single household‐level human‐needs assessment, we first assessed each basic need per person and then classified a household as meeting a particular need if each person in the household had met the need.

The basic need of participation was assessed in Mozambique but not in Kenya due to different approaches used. In the latter, where respondents were solely asked about their membership in organizations, the question was frequently misinterpreted and could not be readily assessed against a participation threshold.

### Exploring How Ecosystem Services Contribute to Needs

In step 5, a group discussion was held with a diversity of key informants at each site to elicit the benefits (ecosystem services) they obtain from the environment. A number of different ecosystem services were identified (Supporting Information). The compiled list of services from these discussions fed into a further 2 (1 male and 1 female) focus groups at each site. In these focus groups, for each of the basic needs, we asked participants which of the ecosystem services contributed to it in that site, why they did, and how important this effect was (1, little importance, to 3, very important). Descriptive quantitative analysis was conducted to elucidate the relative importance of different ecosystem benefits for different needs. We present findings from the 5 ecosystem‐derived benefits that were perceived to be the most important for well‐being across the 8 sites studied.

## Results

### Identifying When Needs are Met

All well‐being criteria described by participants in response to questions about who in the community is doing well or doing badly across sites (step 1) were closely related to different needs identified by the theory of human need (Supporting Information). Certain well‐being criteria mentioned for those doing well or badly could form part of one or a number of different human needs. In Mieze, for example, someone doing very well was described as someone who participates in agricultural activities that involve producing goods for food or for business and therefore contributes to economic or food security (Supporting Information). Conversely, no well‐being criteria were associated with someone doing well or badly with regard to water availability at any site. In Mozambique, other needs such as physical security, respect, autonomy, participation, and relationships were also not linked to any specific needs in certain sites (Supporting Information).

When eliciting indicators of doing well or badly for each need (step 2), focus groups showed substantial variation in their interpretation of what it means to do well (Table 1). However, characteristics of doing badly for each need were consistent at each site. Indicators clustered around thresholds of harm could be categorized according to 1 or 2 broader characteristics. In the case of education, for example (Table 1), indicators of serious harm were similar across sites and included school attendance (in particular due to school or enrollment fees) and scholarly equipment (e.g., books and adequate clothing). Lack of adequate scholarly equipment was thought to prevent children from attending school; therefore, only questions related to being enrolled at school and school attendance were included in the household questionnaire. Participants in Mieze felt that although some in the community were doing badly in terms of education, nobody was in serious harm and therefore no indicators were found to be below the threshold of harm.

**Table 1:** Basic needs related to education and thresholds of harm identified by focus group participants in 8 total sites in Kenya and Mozambique

Mozambique basic needs by site				Kenya basic needs by site			
Vamizi	Lalane	Maringanha	Mieze	Mkwiro	Vanga	Kongowea	Tsunza
studies at high school has uniform has school bag takes lunch to school finish 5th level (primary) has exercise book has shoes for school uses hands to write on does not have uniform	well behaved take good care of their books parents do school enrollment children give up school children do not have lunch children do not have uniform	children go to university parents take children to school by car children take lunch and money to school children have a cell phone have all necessary school equipment children finish secondary school parents take their children to school by motorbike has shoes has school bag study until 7th level (primary) uses hands to write on does not have uniform	children attend high school has all necessary school equipment children go to school do not finish the school level father does not worry about child education children rarely go to school children do not have necessary school equipment or lunch	ability to pay fees children in private schools easy to attend university extra tuition children only attend some years due to school fees reliance to sponsorship/donor children attend government schools	united family good health savings upheld children in private schools fees fully paid full uniforms and stationery no savings no tuition fees not paid children at home most time instead of being in schools continued conflicts	international education system expensive or special schools private schools education to university guaranteed fully paid fees private tuition private schools/ low‐quality education school tuition seasonal financing of children education few teachers and large class sizes no transport to school no equipment free education	education up to university extra tuition extra teachers employed privately children in academy guaranteed employment children in public schools children are very bright have at least one uniform set moderate fee payment persistent problems in schools fees at secondary level no pocket money children drop out at end of primary level insufficient school supplies
**Threshold of harm**							
does not finish primary school	parents do not enroll kids to school	parents do not enroll kids to school		cannot afford school fees lack of food makes children too weak to attend school children drop out of school no family planning	low education interest parents and children drop out of school due to early marriages	children drop out at end of primary level no vision in education irregular attendance to school no tuition cannot afford fees no uniforms	lack of morale for school children cannot afford fees children have no time to study (need to support parents with other duties)

Due to the similarities in indicators clustered around thresholds of harm, similar questions in the survey were asked at each site. In the case of education, a household was considered to be in serious harm if children were not enrolled at school or missed school once a week or more.

Such consistency in indicators around thresholds of harm across sites occurred for most human needs, but not all. When considering water, for example, combinations of answers that determined serious harm or not differed between sites. Unlike other sites, having access to a well in Lalane did not exempt households from being in serious harm because water quality in the well was deemed by the field and research team with extensive knowledge of the sites to be very poor due to its shared use with animals and livestock and proximity to the sea.

### Needs Being Met

**Figure 2:**
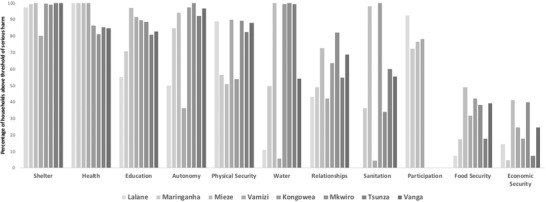
Percentage of households per site above the threshold of serious harm for each basic need in Kenya (black) and Mozambique (gray).

Overall, the level of needs fulfilled was higher in Kenya (mean = 78.5% [SD 11.4]) than in Mozambique (mean = 61.9% [14.2]). We found strong variation in needs fulfillment between sites within a country for some needs, such as sanitation and economic security in Kenya and water, autonomy, and education in Mozambique (Fig. 2). For several needs, however, we found strong similarities among all sites. Nearly all households had their need for shelter, health, and autonomy fulfilled (more information about proportion of needs met and unmet at each site for men and women is available at http://www.espa-spaces.org/resources/spaces-data-explorer/).

### Contribution of Ecosystem Services to Meeting Human Needs

The mentioned ecosystem‐derived benefits across sites were diverse (Supporting Information). Provisioning services were most frequently cited and considered most important, although regulating services, such as provision of shade, and cultural services, such as tourism, were also mentioned. The majority of effects of ecosystem services on well‐being were positive, but some negative examples (e.g., collecting of shells having a negative effect on school attendance) were given. Combining the importance ascribed to each good for different human needs from each focus group allowed us to explore how the surrounding environment contributed to different well‐being domains. The approach taken, however, biases provisioning and cultural services because it emphasizes what people relate to most directly. This is one of the method's strengths because it enables one to explore goods and services that experts may overlook that are important for people in different contexts. However, it can also be a weakness because it may not include more invisible supporting and regulating services.

Although fish and octopus were both perceived as very important for different needs across both countries, in Mozambique a greater importance was attributed to them for certain specific needs, in particular food security, economic security, and relationships (Table 2). Kenyan participants perceived ecosystem services to be more important for a wider range of needs. This was particularly so in the case of mangrove poles and firewood, which were perceived as important for a wider set of needs in Kenya than in Mozambique.

**Table 2:** Combined men's and women's focus groups’ perceived importance of goods (as a percentage of maximum importance that could be attributed) derived from the environment across Kenyan and Mozambican study sites

Country and ecosystem‐derived good	Health	Education	Physical security	Water	Respect	Autonomy	Shelter	Food	Economic security	Participation	Sanitation	Relationships
**Kenya**												
Fish	79	96	58	67	63	92	88	83	88	54	50	88
Octopus	50	58	33	38	38	29	46	54	33	21	33	42
Mangrove poles	46	79	63	25	29	33	83	63	67	25	54	50
Mangrove firewood	33	58	33	17	13	75	29	83	71	38	13	58
Shells	8	25	0	21	13	17	21	8	29	13	21	46
**Mozambique**												
Fish	50	96	21	0	75	71	79	96	96	63	75	96
Octopus	46	75	13	0	54	42	46	79	79	50	50	58
Mangrove poles	0	29	54	0	21	33	79	4	42	25	58	33
Mangrove firewood	4	8	25	0	0	8	0	17	17	4	17	4
Shells	0	54	0	0	29	38	29	67	46	25	33	29

Gender had a strong effect on the perception of ecosystem‐service benefits and their contribution to needs. Women's focus groups perceived mangrove firewood of particular importance to education, due to its role in cooking and hence food and nutrition security of children and importance as a source of income to be used for buying school uniforms. Men, however, perceived mangrove firewood to be predominantly important for physical security because it can be used for self‐defense to protect oneself and one's family in the event of an intruder.

We also found evidence of trade‐offs in the interactions of ecosystem‐service needs. For example, shell picking was perceived to be important for education at most sites by both men and women because income obtained from harvesting and selling shells contributed to school fees, uniforms, and equipment. However, it was also perceived to have a negative effect on the education of girls in Mozambique who regularly miss school at low tides to pick shells.

## Discussion

The human‐needs approach enabled us to characterize the extent and nature of multidimensional poverty based on locally grounded indicators of deprivation to a range of specific needs. Second, it provided a framework to explore how environmental benefits contribute to people meeting their needs. It can therefore help target development interventions toward needs that are least met at each site (Fig. 2), to consider how benefits derived from the environment are making significant contributions to meeting these needs currently, and to monitor and evaluate conservation plans to ensure they have not pushed people into serious harm.

Decision makers could use this approach to consider anticipated impacts of conservation on different ecosystem services and to explore repercussions actions would have on different needs. Octopus, for example, may not be perceived as important for economic security in Kenya and therefore the impacts of conservation interventions such as marine protected areas or gear restrictions that may reduce access to octopus may not be given much weight. Our findings, however, suggest that octopus is important for a range of different needs such as health, education, and food security, which may result in some households no longer meeting these needs and hence experiencing serious harm. This approach may also prove useful when considering the social impacts of large‐scale development policies on removing access to ecosystem services, such as the current situation with the expansion of the oil and gas industry in northern Mozambique.

The multidimensional description of deprivation within communities can also challenge perceptions and open up new avenues for resource management or poverty alleviation. Fishing households, for example, had higher likelihood of meeting income security and education needs but often had lower or no greater chance of meeting other needs such as shelter, sanitation, and food security. This indicates that the higher incomes of fishing households may not translate into relief of multidimensional poverty and may open up avenues to navigate trade‐offs between fishing.

Our results also highlight how actively participating in meetings and interaction with others in a community is deemed important for human needs of respect and relationships and is linked to the threshold of harm for autonomy. This highlighted a window of opportunity for Vamizi, where there is a community‐based marine protected area. Ensuring a broader participation in fisheries decision making around the MPA could improve the number of people meeting these basic needs.

One of the merits and difficulties of this approach is the tension that exists between expert and local views on when a need is met or unmet. The demise of needs thinking in the 1980s can be attributed to the paternalistic attitude surrounding the approach. It was deemed arrogant to lay down what people should regard as a human need (Streeten 1984). The participatory and inclusive process of deriving thresholds in this study helps address that critique; indicators for each need were created during focus groups. However, when deciding on when a need was met or not, some in‐country expert opinions were required. The focus group participants may have adapted to poor conditions and accept conditions that are seriously harmful as simply part of life. This reflects Sen's (2001) concern with adaptive preferences in which people internalize the harshness of their circumstances so that they do not desire what they can never expect to achieve (see also, Clark 2012). In Lalane (rural Mozambique), for example, the majority of households have access to only 2 wells with poor water quality; however, access to safe drinking water was not identified by the focus groups as an issue for the community. This demonstrates the need for an expert view to make sure that the threshold of harm is not set too low by local participants. Future work in these communities, however, could be carried out with the same questionnaire, which would remove the need to replicate steps 1–4 and provide a more rapid needs assessment. With little extra time or monetary cost, the thresholds‐of‐harm questions can be asked as part of social impact surveys that increasingly form a part of many projects.

Although the list of basic human needs does not vary and is universal, the ways in which these are met are context specific and may potentially vary over time. New technological advances or development projects, for example, may provide different means to meeting a basic need. Other changes in a socioecological context such as new environmental pressures or changes in the demography may also affect how needs are met or unmet, complicating the relationship between conservation actions and basic needs. For example, conservation that limits access to a resource may not impact people's needs if this coincides with new accessible and acceptable (or even favorable) ways of meeting that need. Alternatively, basic needs may become unmet in the course of, but not due to, conservation action as a result of concomitant social or ecological changes. Our approach can be used to assess multidimensional deprivation but cannot be used to attribute deprivation to particular causes such as conservation interventions. However, conservationists could adopt or supplement the method to monitor the effects of specific actions.

General improvements in welfare may also lead to reevaluations as to what constitutes meeting a basic need, thus shifting thresholds of harm over time. Despite the potential for thresholds to be context and time specific, our data showed a surprising consistency of thresholds across a range of urban to rural sites in 2 countries, suggesting that thresholds of harm in meeting the most basic needs are relatively consistent across different contexts––even if aspirations may be different in different sites. This supports the use of thresholds as an indicator of deprivation, but they should not be uncritically used over long or transformative periods. It may be prudent to repeat focus groups to check that thresholds remain appropriate. An avenue for future work would be to carry out longitudinal studies to see how these thresholds of harm shift in different contexts and what factors may predict this movement.

Another opportunity to further align poverty reduction and environmental sustainability would be to question solely those below or around the threshold of harm. By understanding how those in serious harm engage with ecosystem services and how these services contribute to their different needs, one can get a more accurate picture of the ecosystem services critical for those most in need rather than for the whole community.

Furthermore, while the needs approach allows a holistic evaluation of multiple dimensions of deprivation, it does not solely consider conservation interventions and their impacts on well‐being. Whether or not harm as a result of missing basic needs is caused or alleviated directly by conservation efforts, people being deprived of their basic needs impose instrumental and moral constraints and responsibilities on conservation organizations. Future work could pay more attention to how people feel about conservation governance, which has been shown by Dawson et al. (2017) to vary independently of more objective measures of well‐being. Thus, our approach could be complimented by an environmental‐justice approach that more explicitly addresses people's experiences of different dimensions of environmental justice.

Our needs approach can be used to identify a context‐specific minimum threshold of human welfare below which a person would incur serious harm of an objective kind. Policies to conserve resources, if poorly designed, can push people into serious harm and vice versa. Currently, although do‐no‐harm conservation sounds like a good principle and ethic to follow, practitioners have little idea of what that means in practice. Using a list of needs helps break down the concept of harm by clearly defining it. This approach also elucidates the link between different needs and ecosystem services. Combining these 2 aspects allows decision makers to ascertain which are the critical ecosystem services for human needs in different contexts. It can also help in monitoring and evaluating the impact of conservation plans so as to ensure that these do not increase the number of people deprived of basic needs. The approach therefore seeks to balance and integrate the frequently competing interests of conservation and development in socialecological systems. As such, it can inform the search for policy or interventions that lead to positive environmental changes that at the very least do not result in serious harm to people.

## Supporting information

The distinct steps followed to assess the level of basic needs (Appendix S1), a focus group guide which explains how to derive the thresholds of harm (Appendix S2), the different ecosystem services identified and their relative contributions to basic needs (Appendix S3), the summary data which identify all well‐being criteria and their associated needs across all sites (Appendix S4), the example of culturally relevant well‐being criteria described by participants in Mieze, Mozambique (Appendix S5), and the frequency of well‐being criteria being discussed at each site (Appendix S6) are available online. The authors are solely responsible for the content and functionality of these materials. Queries (other than absence of the material) should be directed to the corresponding author.

Supporting Information

Supporting Information

Supporting Information

Supporting Information

Supporting Information
