# Supplementary material for: Incorporating basic needs to reconcile poverty and ecosystem services
Source: Conserv Biol. 2018 Nov 20;33(3):655–64. doi: 10.1111/cobi.13209 (PMC7379688; doi:10.1111/cobi.13209)

Process flow for deriving and operationalising basic needs and evaluating them amongst a representative sample of populations and exploring their relationship to different ecosystem services. Shaded boxes indicate empirical fieldwork.


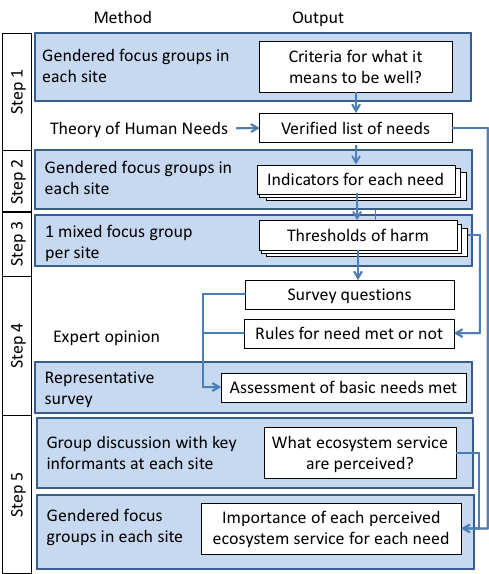

Supplement: Supplementary file 1 — The distinct steps followed to assess the level of basic needs (Appendix S1), a focus group guide which explains how to derive the thresholds of harm (Appendix S2), the different ecosystem services identified and their relative contributions to basic needs (Appendix S3), the summary data which identify all well‐being criteria and their associated needs across all sites (Appendix S4), the example of culturally relevant well‐being criteria described by participants in Mieze, Mozambique (Appendix S5), and the frequency of well‐being criteria being discussed at each site (Appendix S6) are available online. The authors are solely responsible for the content and functionality of these materials. Queries (other than absence of the material) should be directed to the corresponding author. [file COBI-33-655-s001.docx]
