# Supplementary material for: Incorporating basic needs to reconcile poverty and ecosystem services
Source: Conserv Biol. 2018 Nov 20;33(3):655–64. doi: 10.1111/cobi.13209 (PMC7379688; doi:10.1111/cobi.13209)
Supplement: Supplementary file 2 — Supporting Information [file COBI-33-655-s002.docx]

# Focus Groups Guide to Identifying Thresholds of Harm

### What for?

- To identify thresholds of harm for each overall Needs criteria
- Only those indicators below which individuals are thought to be harmed should be used for survey
- By identifying their thresholds of harm, this will then inform the survey questions pertaining to whether basic needs are met.

### Who with?

- 6 people to be selected from the previous 2 Needs Focus Groups. These 6 need to span a number o categories, Male/Female, different livelihoods, different poverty levels if possible.
- Most importantly, they need to be those that were most engaged in the previous focus groups (3 are chosen from each focus group)

### Roles

1. Moderator 1:
   - Introduces the Project and the different members
   - Moderator of whole group for question 1 and 2
   - Moderator for mini-group
   - Summarises and concludes focus group
2. Moderator 2:
   - Puts information on flip charts
   - Assists Moderator 1
   - Pays attention to group dynamics
   - Moderator for mini group
3. Moderator 3
   - Writes information for each individual and their seating arrangement for main focus group and for the mini groups
   - Takes notes of main Focus Group paying attention to important comments, conflicts, body language, interactions
   - Moderator for mini group

### Resources Needed

1. Snacks and Tea
2. Flip Chart Paper
3. Pens
4. 3 recorders
5. Participant Travel Cost money
6. Numbered Stickers

### Step 1:

#### What for?

- To outline the concepts of 'well-being' used by residents
- To develop wellbeing criteria and verify these with the list of universal needs criteria

#### Who with?

Participants should represent a cross-section of the community. Different social, economic, and cultural groups should take part in this exercise. In order of importance for selection criteria. Nine participants selected and two focus groups carried out at each site.

#### Questions and Probes

1. How would you describe a household in this community that is doing well?
   - Think of material things a household might have or lack
   - Think of the sorts of things the household might do
   - Think of how members of the household might be in themselves
   - Think of the things these household members might know
   - Think of the different (kinds of) people members of the household might connect with
2. How would you describe a household in this community that is not doing well?
   - Think of material things a household might have or lack
   - Think of the sorts of things the household might do
   - Think of how members of the household might be in themselves
   - Think of the things these household members might know
   - Think of the different (kinds of) people members of the household might connect with

*Write criteria on flip-chart. Fuse these with the universal criteria list during the tea break. Split the group up into three “mini groups”.*

### Step 2: Questions and Probes

#### What for?

- To identify a series of needs indicators which are contextually relevant

#### Who with?

- Same participants as for step 1

#### Questions and Probes

1. How would you describe a household that is/isn’t economically secure?
   - What material things/assets are associated with less/more economically secure households?
   - What kind of life is associated with an economically stable/unstable household?
2. How would you describe a household that is/is not meeting its needs for sufficient food?
   - What do they eat?
   - How often do they eat?
3. How would you describe a household that has/has not got clean drinking water?
   - Where do they get there water from?
   - How do they get their water
   - Probe?
4. How would you describe a household has/has not got sufficient sanitation?
   - Does it have toilets?
   - Different types of toilets
5. How would you describe a household that has/has not got adequate shelter?
   - Own a house?
   - Type of House?
   - House Characteristics
   - Big enough for family
6. How would you describe a household that has/has not achieved adequate education?
   - Education Levels of the household
   - Number of people attending or attended school (primary, then secondary etc..)
   - Type of education (good or bad)
7. How would you describe a household that has/has not got adequate health care?
   - How often ill/sick
   - Access to health care
   - Are treatments pursued? By who?
   - Type of treatment, by who?
   - Does this depend on severity of illness?
8. How would you describe a household that is/is not physically secure?
   - Fear of attack
   - Been attacked
   - Security within community
9. How would you describe a household that can make decisions for itself
   - Do people come for advice?
   - To what extent can you make and realise your own decisions?
10. How would you describe a household that has/has not got good emotional relationships?
    - Number of good friends outside the family
    - Satisfaction of relationships within household, with neighbours, with others in village and outsiders
11. How would you describe a household that does/doesn’t participates in society?
    - Number of participatory activities household members have been a part of ?
    - What activities do they participate in?
    - How often do they participate?
12. How would you describe a household that is/isn’t respected by the community?
    - What level of leadership they have in the community
    - Does the community consult them in any issues
    - What types of issues are they involved in?

*Remember that there may be more contextual criteria to add to this list, depending on what is answered in questions 1 and 2. The probes used for each of these needs may differ and could be made more contextually relevant by incorporating answers from step 1.*

### Step 3: Questions and Probes

#### What for?

- To identify thresholds of harm for each overall Needs criteria
- Only those indicators below which individuals are thought to be harmed should be used for survey
- By identifying their thresholds of harm, this will then inform the survey questions pertaining to whether basic needs are met.

#### Who with?

- 6 people will be selected from the previous 2 Needs Focus Groups. These 6 need to span a number of categories, gender, different livelihoods, different levels of wealth.
- Most importantly, they need to be those that were most engaged in the previous focus groups (3 are chosen from each focus group)

#### Questions and Probes

*For each human need, list their different indicators identified from step 2, place them on a line and organise them as to whether these are indicative of poor, rich or non poor households. Once this is done, get participants to determine at which point for each criteria, the household would suffer serious harm.*

Now that we have a total list of needs indicators specific to this community, which do you think is the order of importance.

- - If an individual in this community had absolutely nothing and had to start re-building his/her life. Which of these needs would he/her seek to accomplish first, second, third etc…

*Make sure that these are written up on the board and there is a drawing associated with each. They are then given numbered stickers to place on the flip chart.*
