# Supplementary material for: Incorporating basic needs to reconcile poverty and ecosystem services
Source: Conserv Biol. 2018 Nov 20;33(3):655–64. doi: 10.1111/cobi.13209 (PMC7379688; doi:10.1111/cobi.13209)
Supplement: Supplementary file 3 — Supporting Information [file COBI-33-655-s003.docx]

|  | Health | Education | Physical Security | Water | Respect | Autonomy | Shelter | Food | Economic Security | Participation | Sanitation | Relationships | Average Importance |
| --- | --- | --- | --- | --- | --- | --- | --- | --- | --- | --- | --- | --- | --- |
| Fish | 65 | 96 | 40 | 33 | 69 | 81 | 83 | 90 | 92 | 58 | 63 | 92 | 72 |
| Octopus | 48 | 67 | 23 | 19 | 46 | 35 | 46 | 67 | 56 | 35 | 42 | 50 | 44 |
| Mangrove Poles | 23 | 54 | 58 | 13 | 25 | 33 | 81 | 33 | 54 | 25 | 56 | 42 | 41 |
| Mangrove Firewood | 19 | 33 | 29 | 8 | 6 | 42 | 15 | 50 | 44 | 21 | 15 | 31 | 26 |
| Shells | 4 | 40 | 0 | 10 | 21 | 27 | 25 | 38 | 38 | 19 | 27 | 38 | 24 |
| Honey | 46 | 15 | 13 | 4 | 15 | 13 | 8 | 31 | 27 | 6 | 0 | 10 | 16 |
| Tourism | 8 | 23 | 10 | 15 | 15 | 2 | 19 | 8 | 17 | 4 | 17 | 21 | 13 |
| Mangrove Medicine | 60 | 4 | 15 | 0 | 19 | 2 | 2 | 10 | 10 | 4 | 0 | 13 | 12 |
| Mangrove Toilet | 10 | 0 | 13 | 6 | 19 | 0 | 6 | 0 | 0 | 13 | 27 | 10 | 9 |
| Shade | 25 | 10 | 10 | 0 | 4 | 2 | 6 | 0 | 0 | 17 | 6 | 6 | 7 |
| Reference Point | 6 | 6 | 27 | 0 | 8 | 6 | 4 | 2 | 6 | 8 | 0 | 13 | 7 |
| Dye | 0 | 4 | 0 | 0 | 2 | 6 | 8 | 4 | 19 | 6 | 6 | 8 | 5 |
| Brewing | 6 | 2 | 4 | 2 | 4 | 0 | 0 | 0 | 0 | 4 | 0 | 10 | 3 |

Ecosystem services and their relative contribution to basic needs (expressed as a proportion of maximum importance that could be attributed)
