# Supplementary material for: Incorporating basic needs to reconcile poverty and ecosystem services
Source: Conserv Biol. 2018 Nov 20;33(3):655–64. doi: 10.1111/cobi.13209 (PMC7379688; doi:10.1111/cobi.13209)
Supplement: Supplementary file 5 — Supporting Information [file COBI-33-655-s005.docx]

Culturally relevant wellbeing criteria described by participants in one focus group in Mieze Mozambique and their perceived links to a universal list of needs.

| Wellbeing Criteria | Doing well or badly | Description | Relevant Need from THN |
| --- | --- | --- | --- |
| Does Agriculture | Well | Produces agricultural products for food and do business | Food Security, Economic Security |
| Sleeps well | Well | Eating well and feeling safe within the house helps sleep | Food security, Physical security |
| Has money | Well | Has money for all basics needs at home. | Economic Security |
| Has Food | Well | Has use of many ingredients | Food Security |
| Is healthy | Well | Is healthy to perform daily activities. | Health |
| Has a good house | Well | Has a complete home with furniture and zinc roof | Shelter |
| School | Well | Children go to school with lunch. | Education |
| Is Ambitious | Well | Thinks about progressing own life | Autonomy |
| Is Honest | Well | Honesty and kindness with regards to the poor is highly commended by the community | Relationships, Respect |
| Has Electricity | Well | Having assets in the home such as electricity | Shelter |
| No food worries | Well | Does not need to think much to feed family | Food Security |
| Goes to church | Well | Goes to church to pray. | Participation |
| Drinking | Badly | Drinks a lot of alcohol and is seen badly by others | Respect |
| No responsibility | Badly | Is not responsible with money and is frowned upon by community for not using money effectively | Respect |
| No house | Badly | Has no house of their own | Shelter |
| No objectives | Badly | Does not fulfil their own objectives | Autonomy |
| Has bad luck | Badly | Is often unlucky and feels they cannot change their situation | Autonomy |
| Is a thief | Badly | Takes other people’s things to sell | Economic Security, Respect, Relationships |
| No education | Badly | Does not make children go to school | Education |
| Lazy | Badly | Has capacity to work but does not do it | Autonomy |
| Irresponsible | Badly | Uses any money for drinks rather than for shelter and food | Respect |
| Jealous | Badly | Thinks badly of others and hopes they become unsuccessful | Relationships |
