# Supplementary material for: Incorporating basic needs to reconcile poverty and ecosystem services
Source: Conserv Biol. 2018 Nov 20;33(3):655–64. doi: 10.1111/cobi.13209 (PMC7379688; doi:10.1111/cobi.13209)
Supplement: Supplementary file 6 — Supporting Information [file COBI-33-655-s006.docx]

Frequency of focus groups (max 2) which alluded to different human needs when participants were asked to describe who is living “well” and “badly” at each site

| **Country** | **Site** | **Health** | **Education** | **Physical Security** | **Water** | **Respect** | **Autonomy** | **Shelter** | **Food** | **Economic Security** | **Participation** | **Sanitation** | **Relationships** |
| --- | --- | --- | --- | --- | --- | --- | --- | --- | --- | --- | --- | --- | --- |
| Mozambique | Vamizi | 1 | 2 | 0 | 0 | 0 | 0 | 2 | 2 | 2 | 0 | 1 | 0 |
|  | Lalane | 2 | 2 | 0 | 0 | 2 | 2 | 2 | 2 | 2 | 0 | 1 | 0 |
|  | Maringanha | 1 | 1 | 0 | 0 | 0 | 0 | 1 | 1 | 1 | 0 | 1 | 1 |
|  | Mieze | 1 | 2 | 2 | 0 | 1 | 2 | 2 | 2 | 2 | 1 | 2 | 2 |
| Kenya | Mkwiro | 2 | 2 | 2 | 0 | 2 | 1 | 2 | 2 | 2 | 2 | 2 | 2 |
|  | Vanga | 2 | 2 | 2 | 0 | 2 | 2 | 2 | 2 | 2 | 2 | 2 | 2 |
|  | Kongowea | 2 | 2 | 2 | 0 | 2 | 2 | 2 | 2 | 2 | 2 | 2 | 2 |
|  | Tsunza | 2 | 2 | 2 | 0 | 2 | 2 | 2 | 2 | 2 | 2 | 2 | 2 |
